# Supplementary material for: Seasonal and Geographic Variation in Alkaloid Content of Kratom (Mitragyna speciosa (Korth.) Havil.) from Thailand
Source: Plants (Basel). 2023 Feb 19;12(4):949. doi: 10.3390/plants12040949 (PMC9966779; doi:10.3390/plants12040949)
Supplement: Supplementary file 1 [file plants-12-00949-s001.zip › plants-2208871-supplementary.pdf]

Supplementary Figures

Seasonal and Geographic Variation in Alkaloid Content of Kratom (*Mitragyna speciosa* (Korth.) Havil.) from Thailand

Narumon Sengnon [1], Phanita Vonghirundecha [1], Wiraphon Chaichan [2], Thaweesak Juengwatanatrakul [3], Jumpen Onthong[4], Pongmanat Kitprasong[4], Somchai Sriwiriyajan [5], Somsmorn Chittrakarn [5], Supattra Limsuwanchote [5], and Juraithip Wungsintaweekul [1,*]

[1] Department of Pharmacognosy and Pharmaceutical Botany, Faculty of Pharmaceutical Sciences, Prince of Songkla University, Hat Yai campus, Songkhla 90112, Thailand
[2] Narcotic Crops Survey and Monitoring Institute, Office of the Narcotics Control Board, City Hall, Muang, Chiang Mai 50303, Thailand
[3] Faculty of Pharmaceutical Sciences, Ubon Ratchathani University, Warinchamrab District, Ubon Ratchathani 34190, Thailand
[4] Agricultural Innovation and Management Division, Faculty of Natural Resources, Prince of Songkla University, Hat Yai campus, Songkhla 90110, Thailand
[5] Division of Health and Applied Sciences, Faculty of Science, Prince of Songkla University, Hat Yai campus, Songkhla 90110, Thailand
* Correspondence: juraithip.w@psu.ac.th; Tel.: +66-815980868

Figure S1. Protocol of kratom collection suggested by the Narcotic Crop Survey and Monitoring Institute (NCS), the Office of Narcotic Control Board (ONCB).

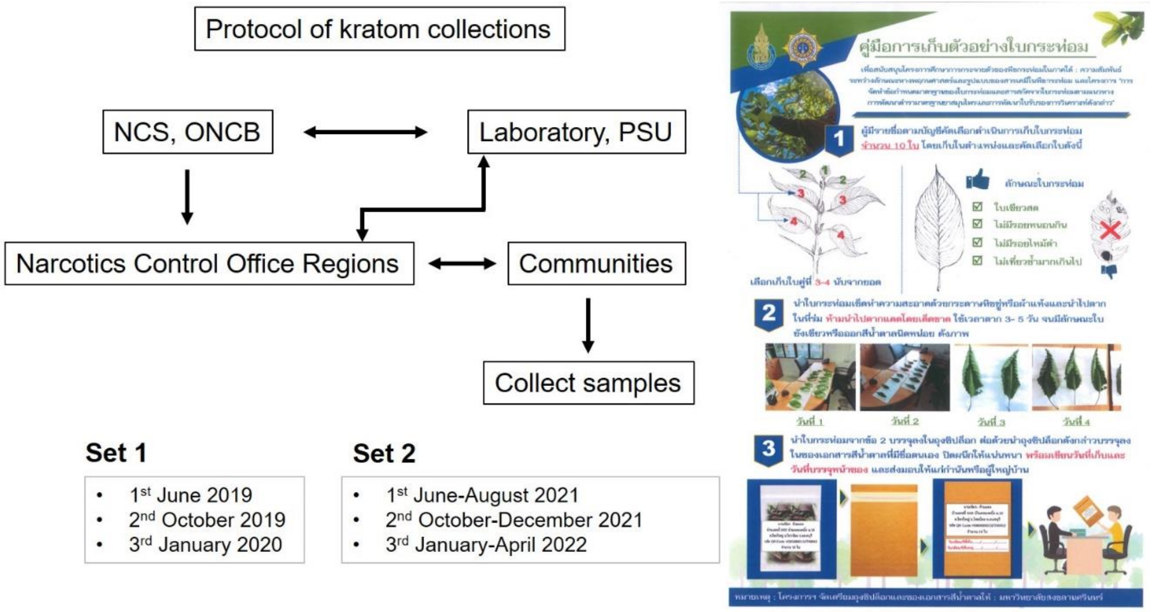

Figure S2.

Kratom specimens from Nam Phu subdistrict, Ban Na San, Surat Thani, Thailand, deposited in the PSU herbarium, Department of Biology, Faculty of Science, Prince of Songkla University, Songkhla, Thailand.

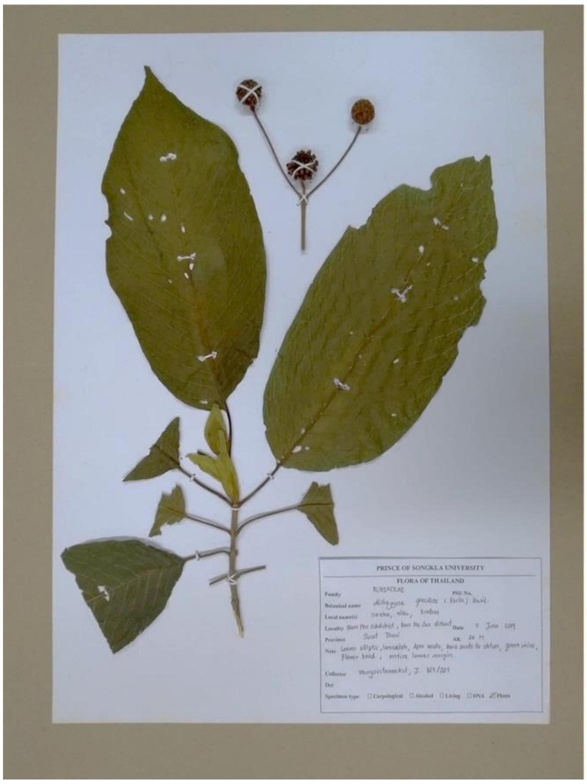

PRINCE OF SONGKLA UNIVERSITY

FLORA OF THAILAND

Family Rubiaceae **PSU No.** N1/001

Botanical name *Mitragyna speciosa* (Korth.) Havil.

Local name(s) กระท่อม, ท่อม, kratom

Locality M 1 Nam Phu Subdistrict, Ban Na San **Date** 7 June 2019

Province Surat Thani **Alt.** 24 m.

Note: leaves opposite, elliptic, lanceolate, apex acute, base acute to obtuse, green vein, flower head, entire leaf margin

Collector Wungsintaweekul, J.

Specimen type ☐ **Carpological** ☐ **Alcohol** ☐ **Living** ☐ **DNA** ☑ **Photo**

Figure S2 (continue)

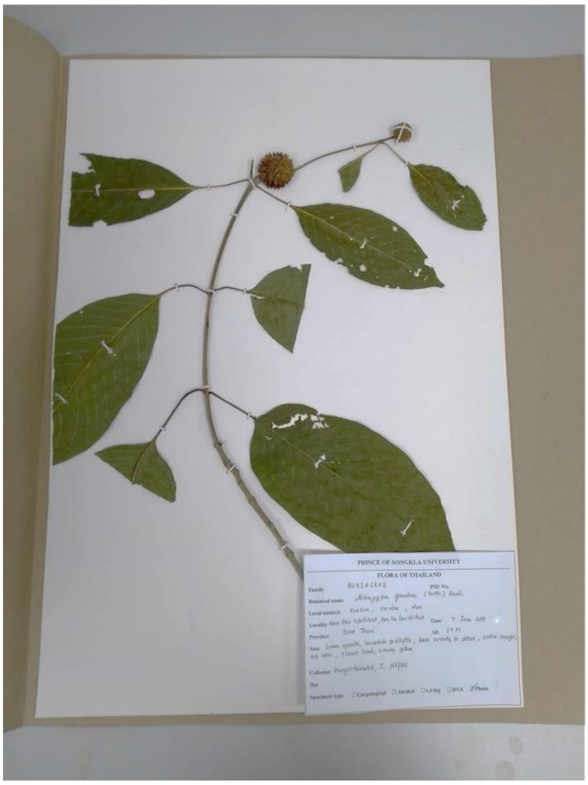

PRINCE OF SONGKLA UNIVERSITY

FLORA OF THAILAND

Family Rubiaceae **PSU No.** N5/001

Botanical name *Mitragyna speciosa* (Korth.) Havil.

Local name(s) กระท่อม, ท่อม, kratom

Locality M5 Nam Phu Subdistrict, Ban Na San **Date** 7 June 2019

Province Surat Thani **Alt.** 27 m.

Note: leaves opposite, elliptic, lanceolate, apex acute, base cuneate to obtuse, red vein, flower head, entire leaf margin

Collector Wungsintaweekul, J.

Specimen type ☐ **Carpological** ☐ **Alcohol** ☐ **Living** ☐ **DNA** ☑ **Photo**

Figure S2 (continue)

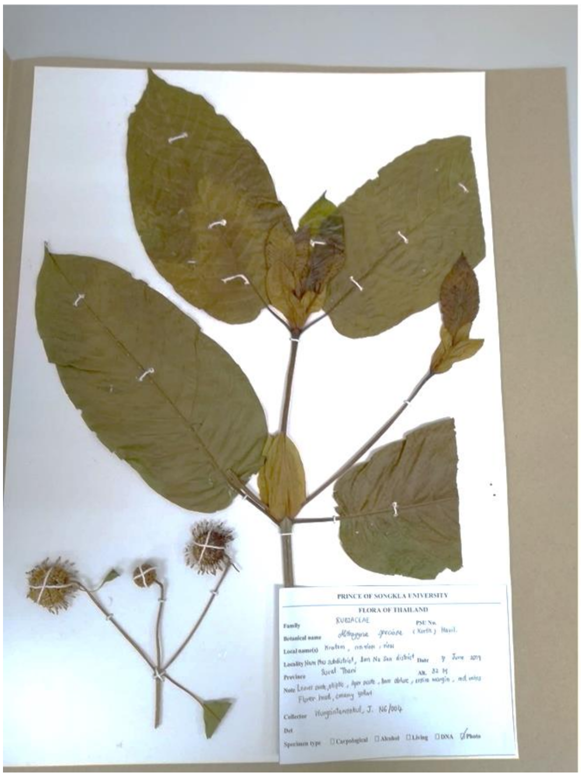

PRINCE OF SONGKLA UNIVERSITY

FLORA OF THAILAND

Family Rubiaceae **PSU No.** N6/004

Botanical name *Mitragyna speciosa* (Korth.) Havil.

Local name(s) กระท่อม, ท่อม, kratom

Locality M 6 Nam Phu Subdistrict, Ban Na San **Date** 7 June 2019

Province Surat Thani **Alt.** 32 m.

Note: leaves opposite, ovate, apex acute, base obtuse, red vein, flower head, entire leaf margin

Collector Wungsintaweekul, J.

Specimen type ☐ **Carpological** ☐ **Alcohol** ☐ **Living** ☐ **DNA** ☑ **Photo**

Figure S2 (continue)

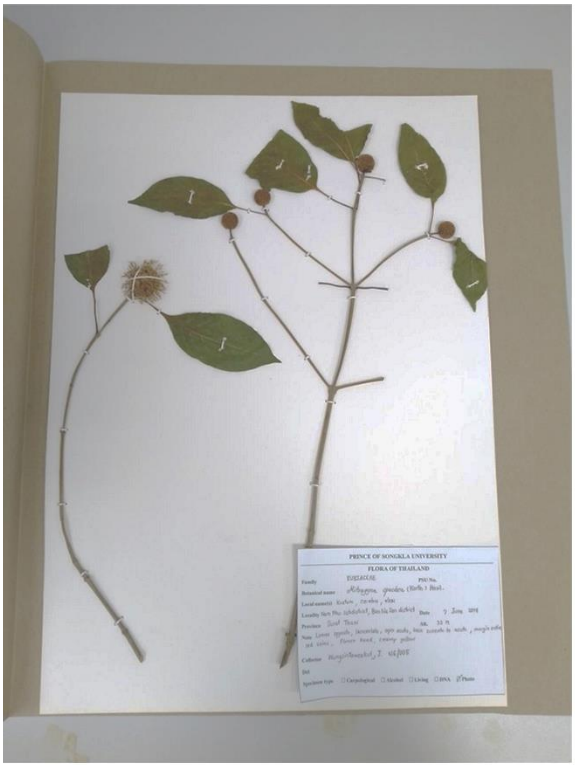

PRINCE OF SONGKLA UNIVERSITY

FLORA OF THAILAND

Family Rubiaceae **PSU No.** N6/005

Botanical name *Mitragyna speciosa* (Korth.) Havil.

Local name(s) กระท่อม, ท่อม, kratom

Locality M 6 Nam Phu Subdistrict, Ban Na San **Date** 7 June 2019

Province Surat Thani **Alt.** 33 m.

Note: leaves opposite, lanceolate, apex acute, base cuneate, red and green vein, flower head, entire leaf margin

Collector Wungsintaweekul, J.

Specimen type ☐ **Carpological** ☐ **Alcohol** ☐ **Living** ☐ **DNA** ☑ **Photo**

Figure S2 (continue)

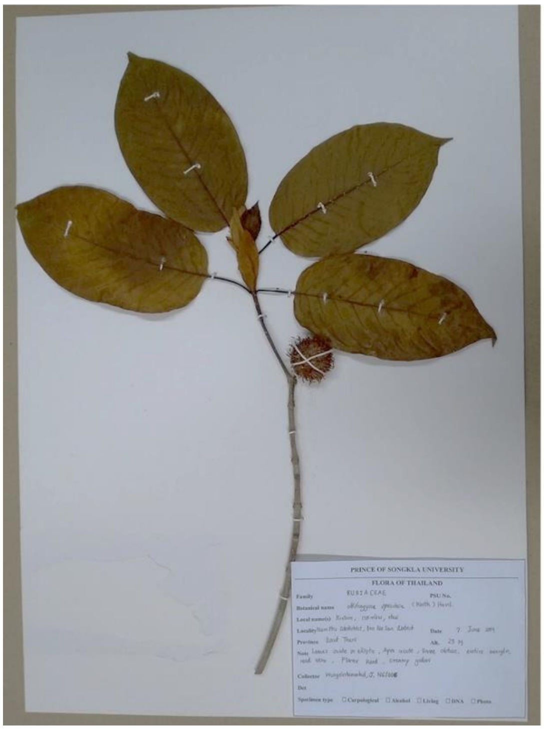

PRINCE OF SONGKLA UNIVERSITY

FLORA OF THAILAND

Family Rubiaceae **PSU No.** N6/006

Botanical name *Mitragyna speciosa* (Korth.) Havil.

Local name(s) กระท่อม, ท่อม, kratom

Locality M 6 Nam Phu Subdistrict, Ban Na San **Date** 7 June 2019

Province Surat Thani **Alt.** 23 m.

Note: leaves opposite, ovate, elliptic, apex acute, base obtuse, red vein, flower head, entire leaf margin

Collector Wungsintaweekul, J.

Specimen type ☐ **Carpological** ☐ **Alcohol** ☐ **Living** ☐ **DNA** ☑ **Photo**

Figure S3. The isolation protocol and chemical characteristics for mitragynine (MG), paynantheine (PAY), and speciogynine (SG).

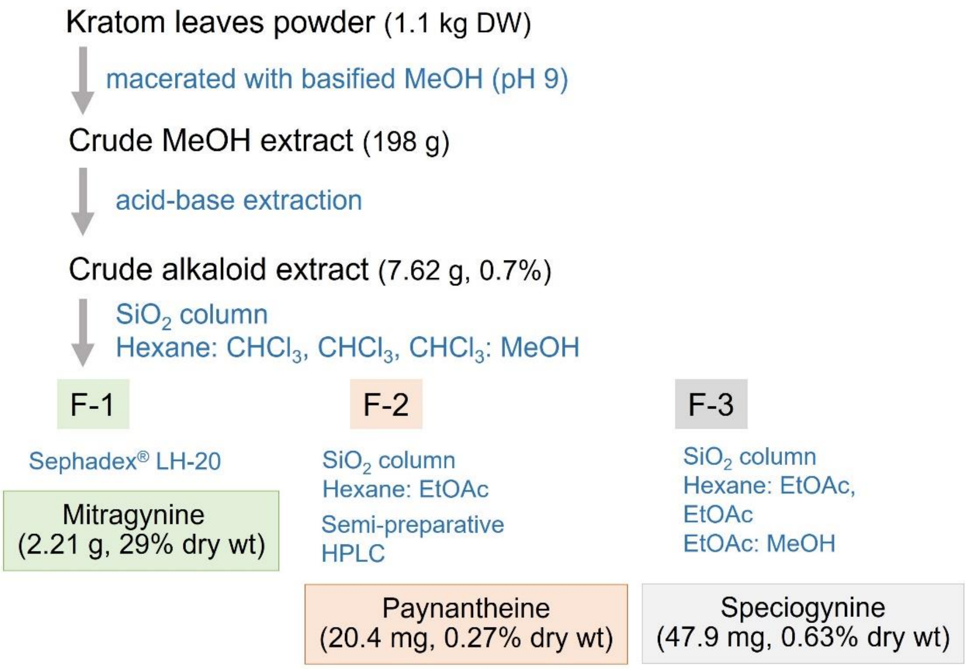

Mitragynine: see in [36].

Paynantheine: a pale yellowish amorphous solid; ^{1}H-NMR (500 MHz, $CDCl_3$) δ: 7.70 (1H, br-s, N-H), 7.31 (1H, s, H-17), 6.98 (1H, dd, J = 8.01, 7.77 Hz, H-11), 6.87 (1H, dd, J = 8.00, 0.69 Hz, H-12), 6.45 (1H, d, J = 7.77 Hz, H-10), 5.55 (1H, dd, J = 17.8, 9.83 Hz, H-19), 5.00 (1H, dd, J = 17.15, 1.38 Hz, H-18), 4.94 (1H, dd, J = 10.29, 2.06 Hz, H-18), 3.85 (3H, s, 9-OCH_3), 3.76 (3H, s, 17-OCH_3), 3.67 (3H, s, 22-OCH_3), 3.30 (1H, br-d, J = 10.51 Hz, H-3), 3.17 (1H, m, H-5), 3.07-2.97 (4H, m, H-6, H-20, H-21), 2.74 (1H, td, J = 11.66, 3.66 Hz, H-15), 2.58 (1H, td, J = 11.44, 4.34 Hz, H-5), 2.27 (1H, t, J = 10.75 Hz, H-21), 2.10 (1H, br-q, J = 11.89 Hz, H-14), 1.95 (1H, br-d, J = 12.35 Hz, H-14); ^{13}C-NMR (125 MHz, $CDCl_3$) δ: 169.3 (C-22), 159.9 (C-17), 154.5 (C-9), 139.3 (C-19), 137.3 (C-13), 132.9 (C-2), 121.9 (C-11), 117.5 (C-8), 115.6 (C-18), 110.1 (C-16), 107.8 (C-7), 104.2 (C-12), 99.7 (C-10), 61.6 (17-OCH_3), 60.8 (C-21), 59.9 (C-3), 55.3 (9-OCH_3), 53.1 (C-5), 51.3 (22-OCH_3), 42.7 (C-20), 38.4 (C-15), 33.3 (C-14), 23.6 (C-6); EI-MS m/z 396 (M^+).

Speciogynine: a colorless crystal; ^{1}H-NMR (500 MHz, $CDCl_3$) δ: 7.68 (1H, br-s, N-H), 7.34 (1H, br-s, H-17), 6.97 (1H, dd, J = 8.22, 7.78 Hz, H-11), 6.86 (1H, d, J = 7.77 Hz, H-12), 6.44 (1H, d, J = 7.77 Hz, H-10), 3.85 (3H, s, 9-OCH_3), 3.71 (6H, br-s, 17-OCH_3, 22-OCH_3), 3.19 (2H, m, H-6, H-21), 3.15 (1H, m, H-3), 3.07 (1H, br, H-5), 3.00 (1H, br-d, J = 15.1 Hz, H-6), 2.60-2.58 (2H, m, H-5, H-20), 2.26 (1H, br, H-15), 2.06 (1H, br, H-21), 1.95 (1H, br, H-14), 1.86 (1H, br, H-14), 1.40 (1H, br, H-19), 1.02 (1H, br, H-19), 0.84 (3H, t, J = 7.32, 7.5 Hz, H-18); ^{13}C-NMR (125 MHz, $CDCl_3$) δ: 169.4 (C-22), 159.9 (C-17), 154.5 (C-9), 137.3 (C-13), 132.9 (C-2), 121.8 (C-11), 117.5 (C-8), 111.6 (C-16), 107.7 (C-7), 104.2 (C-12), 99.7 (C-10), 61.7 (17-OCH_3), 60.9 (C-21), 60.3 (C-3), 55.3 (9-OCH_3), 53.6 (C-5), 51.6 (22-OCH_3), 39.9 (C-20), 38.8 (C-15), 33.7 (C-14), 24.4 (C-19), 23.7 (C-6), 11.4 (C-18); EI-MS m/z 398 (M^+).

Supplementary Tables

Seasonal and Geographic Variation in Alkaloid Content of Kratom [*Mitragyna speciosa* (Korth.) Havil.] from Thailand

Narumon Sengnon [1], Phanita Vonghirundecha [1], Wiraphon Chaichan [2], Thaweesak Juengwatanatrakul [3], Jumpen Onthong [4], Pongmanat Kitprasong [4], Somchai Sriwiriyajan [5], Somsmorn Chittrakarn [5], Supattra Limsuwanchote [5], and Juraithip Wungsintaweekul [1,*]

1 Department of Pharmacognosy and Pharmaceutical Botany, Faculty of Pharmaceutical Sciences, Prince of Songkla University, Hat Yai campus, Songkhla 90112, Thailand
2 Narcotic Crops Survey and Monitoring Institute, Office of the Narcotics Control Board, City Hall, Muang, Chiang Mai 50303, Thailand
3 Faculty of Pharmaceutical Sciences, Ubon Ratchathani University, Warinchamrab District, Ubon Ratchathani 34190, Thailand
4 Agricultural Innovation and Management Division, Faculty of Natural Resources, Prince of Songkla University, Hat Yai campus, Songkhla 90110, Thailand
5 Division of Health and Applied Sciences, Faculty of Science, Prince of Songkla University, Hat Yai campus, Songkhla 90110, Thailand
* Correspondence: juraithip.w@psu.ac.th; Tel.: +66-815980868

Table S1. Set 1 of samples collected from Nam Phu subdistrict, Ban Na San, Surat Thani, Thailand from June 2019-January 2020.

Province	District	Subdistrict	Village [a]	Sample code [b]	QR code [c]	Leaf vein color
Surat Thani	Ban Na San	Nam Phu	M1	M1-1	H0000011480T00001	green
Surat Thani	Ban Na San	Nam Phu	M1	M1-2	H0000011511T00001	green
Surat Thani	Ban Na San	Nam Phu	M2	M2-1	H0000010469T00002	red
Surat Thani	Ban Na San	Nam Phu	M3	M3-1	H0000010727T00001	red
Surat Thani	Ban Na San	Nam Phu	M3	M3-2	n.d.	green
Surat Thani	Ban Na San	Nam Phu	M3	M3-3	H0000010696T00001	red
Surat Thani	Ban Na San	Nam Phu	M3	M3-4	H0000010765T00001	red
Surat Thani	Ban Na San	Nam Phu	M3	M3-5	H0000010700T00002	red
Surat Thani	Ban Na San	Nam Phu	M4	M4-1	H0000010418T00001	green
Surat Thani	Ban Na San	Nam Phu	M4	M4-2	H0000010488T00002	green
Surat Thani	Ban Na San	Nam Phu	M4	M4-3	H0000010432T00001	green
Surat Thani	Ban Na San	Nam Phu	M4	M4-4	H0000010500T00001	green
Surat Thani	Ban Na San	Nam Phu	M4	M4-5	H0000010429T00002	green

Surat Thani	Ban Na San	Nam Phu	M4	M4-6	H0000010538T00002	green
Surat Thani	Ban Na San	Nam Phu	M4	M4-7	H0000010516T00001	green
Surat Thani	Ban Na San	Nam Phu	M5	M5-1	H0000010483T00003	red
Surat Thani	Ban Na San	Nam Phu	M5	M5-2	H0000010504T00001	green
Surat Thani	Ban Na San	Nam Phu	M5	M5-3	H0000010596T00003	green
Surat Thani	Ban Na San	Nam Phu	M5	M5-4	H0000010523T00001	green
Surat Thani	Ban Na San	Nam Phu	M5	M5-5	H0000010580T00002	red
Surat Thani	Ban Na San	Nam Phu	M5	M5-6	H0000010510T00004	green
Surat Thani	Ban Na San	Nam Phu	M5	M5-7	H0000010525T00003	green
Surat Thani	Ban Na San	Nam Phu	M5	M5-8	H0000010572T00001	green
Surat Thani	Ban Na San	Nam Phu	M5	M5-9	H0000010452T00004	red
Surat Thani	Ban Na San	Nam Phu	M5	M5-10	H0000010482T00006	green
Surat Thani	Ban Na San	Nam Phu	M6	M6-1	H0000010345T00003	green
Surat Thani	Ban Na San	Nam Phu	M6	M6-2	H0000010345T00002	red
Surat Thani	Ban Na San	Nam Phu	M6	M6-3	H0000010293T00002	green
Surat Thani	Ban Na San	Nam Phu	M6	M6-4	H0000010293T00001	green
Surat Thani	Ban Na San	Nam Phu	M6	M6-5	H0000010219T00001	red
Surat Thani	Ban Na San	Nam Phu	M6	M6-6	H0000010219T00002	green
Surat Thani	Ban Na San	Nam Phu	M6	M6-7	H0000010290T00001	green
Surat Thani	Ban Na San	Nam Phu	M6	M6-8	H0000010339T00003	green
Surat Thani	Ban Na San	Nam Phu	M6	M6-9	H0000010275T00001	green
Surat Thani	Ban Na San	Nam Phu	M6	M6-10	H0000010371T00003	red
Surat Thani	Ban Na San	Nam Phu	M6	M6-11	H0000010269T00003	green
Surat Thani	Ban Na San	Nam Phu	M6	M6-12	H0000010322T00001	red
Surat Thani	Ban Na San	Nam Phu	M6	M6-13	H0000010366T00001	red
Surat Thani	Ban Na San	Nam Phu	M1	M1-3	H0000011525T00001	green
Surat Thani	Ban Na San	Nam Phu	M1	M1-4	H0000011596T00003	green
Surat Thani	Ban Na San	Nam Phu	M1	M1-5	H0000011527T00002	green
Surat Thani	Ban Na San	Nam Phu	M1	M1-6	H0000011590T00001	green
Surat Thani	Ban Na San	Nam Phu	M1	M1-7	H0000011467T00003	red

Surat Thani	Ban Na San	Nam Phu	M1	M1-8	H0000011505T00001	green
Surat Thani	Ban Na San	Nam Phu	M1	M1-9	H0000010547T00002	red
Surat Thani	Ban Na San	Nam Phu	M1	M1-10	H0000011592T00003	green
Surat Thani	Ban Na San	Nam Phu	M2	M2-2	H0000010623T00003	red
Surat Thani	Ban Na San	Nam Phu	M2	M2-3	H0000010449T00005	green
Surat Thani	Ban Na San	Nam Phu	M2	M2-4	H0000010620T00003	green
Surat Thani	Ban Na San	Nam Phu	M2	M2-5	H0000010489T00002	green
Surat Thani	Ban Na San	Nam Phu	M2	M2-6	H0000010662T00016	green
Surat Thani	Ban Na San	Nam Phu	M2	M2-7	H0000010656T00001	green
Surat Thani	Ban Na San	Nam Phu	M2	M2-8	H0000010659T00005	red
Surat Thani	Ban Na San	Nam Phu	M2	M2-9	H0000010610T00003	red
Surat Thani	Ban Na San	Nam Phu	M2	M2-10	H0000010613T00001	red
Surat Thani	Ban Na San	Nam Phu	M3	M3-6	H0000010708T00002	red
Surat Thani	Ban Na San	Nam Phu	M3	M3-7	H0000010767T00003	green
Surat Thani	Ban Na San	Nam Phu	M3	M3-8	H0000010765T00002	red
Surat Thani	Ban Na San	Nam Phu	M3	M3-9	H0000010713T00002	red
Surat Thani	Ban Na San	Nam Phu	M3	M3-10	H0000010723T00002	red
Surat Thani	Ban Na San	Nam Phu	M3	M3-11	H0000010731T00002	green
Surat Thani	Ban Na San	Nam Phu	M3	M3-12	H0000010743T00001	green
Surat Thani	Ban Na San	Nam Phu	M3	M3-13	H0000010703T00001	green
Surat Thani	Ban Na San	Nam Phu	M4	M4-8	H0000010383T00001	red
Surat Thani	Ban Na San	Nam Phu	M4	M4-9	H0000010551T00001	green
Surat Thani	Ban Na San	Nam Phu	M4	M4-10	H0000010516T00001	red
Surat Thani	Ban Na San	Nam Phu	M4	M4-11	H0000010481T00001	green
Surat Thani	Ban Na San	Nam Phu	M4	M4-12	H0000010530T00001	red
Surat Thani	Ban Na San	Nam Phu	M5	M5-11	H0000010450T00001	green
Surat Thani	Ban Na San	Nam Phu	M5	M5-12	H0000010523T00001	red
Surat Thani	Ban Na San	Nam Phu	M5	M5-13	H0000010444T00002	green
Surat Thani	Ban Na San	Nam Phu	M5	M5-14	H0000010452T00003	green
Surat Thani	Ban Na San	Nam Phu	M5	M5-15	H0000010502T00003	green

Surat Thani	Ban Na San	Nam Phu	M5	M5-16	H0000010568T00002	green
Surat Thani	Ban Na San	Nam Phu	M5	M5-17	H0000010444T00001	green
Surat Thani	Ban Na San	Nam Phu	M6	M6-14	H0000010357T00003	green
Surat Thani	Ban Na San	Nam Phu	M6	M6-15	H0000010324T00001	red
Surat Thani	Ban Na San	Nam Phu	M6	M6-16	H0000010339T00003	red
Surat Thani	Ban Na San	Nam Phu	M6	M6-17	H0000010241T00003	red
Surat Thani	Ban Na San	Nam Phu	M6	M6-18	H0000010323T00001	green
Surat Thani	Ban Na San	Nam Phu	M6	M6-19	H0000010267T00005	green
Surat Thani	Ban Na San	Nam Phu	M6	M6-20	H0000010347T00001	green
Surat Thani	Ban Na San	Nam Phu	M6	M6-21	H0000010322T00001	red
Surat Thani	Ban Na San	Nam Phu	M1	M1-11	H0000010292T00003	green
Surat Thani	Ban Na San	Nam Phu	M1	M1-12	n.d.	green
Surat Thani	Ban Na San	Nam Phu	M1	M1-13	H0000011590T00001	green
Surat Thani	Ban Na San	Nam Phu	M1	M1-14	H0000011592T00003	red
Surat Thani	Ban Na San	Nam Phu	M1	M1-15	H0000011467T00002	green
Surat Thani	Ban Na San	Nam Phu	M1	M1-16	H0000011607T00003	green
Surat Thani	Ban Na San	Nam Phu	M1	M1-17	H0000011505T00001	green
Surat Thani	Ban Na San	Nam Phu	M2	M2-11	H0000010623T00003	green
Surat Thani	Ban Na San	Nam Phu	M2	M2-12	H0000010648T00027	green
Surat Thani	Ban Na San	Nam Phu	M2	M2-13	H0000010620T00003	red
Surat Thani	Ban Na San	Nam Phu	M2	M2-14	H0000010662T00016	green
Surat Thani	Ban Na San	Nam Phu	M2	M2-15	H0000010656T00001	red
Surat Thani	Ban Na San	Nam Phu	M2	M2-16	H0000010659T00005	red
Surat Thani	Ban Na San	Nam Phu	M2	M2-17	n.d.	red
Surat Thani	Ban Na San	Nam Phu	M2	M2-18	H0000010658T00006	red
Surat Thani	Ban Na San	Nam Phu	M2	M2-19	H0000010661T00001	green
Surat Thani	Ban Na San	Nam Phu	M2	M2-20	H0000010621T00004	red
Surat Thani	Ban Na San	Nam Phu	M2	M2-21	H0000010613T00001	red
Surat Thani	Ban Na San	Nam Phu	M2	M2-22	H0000011501T00001	red
Surat Thani	Ban Na San	Nam Phu	M2	M2-23	H0000010413T00001	green

Surat Thani	Ban Na San	Nam Phu	M2	M2-24	H0000010625T00003	red
Surat Thani	Ban Na San	Nam Phu	M2	M2-25	H0000010619T00005	red
Surat Thani	Ban Na San	Nam Phu	M2	M2-26	H0000010641T00001	red
Surat Thani	Ban Na San	Nam Phu	M2	M2-27	H0000010669T00005	red
Surat Thani	Ban Na San	Nam Phu	M2	M2-28	H0000010655T00002	red
Surat Thani	Ban Na San	Nam Phu	M3	M3-14	H0000010708T00002	green
Surat Thani	Ban Na San	Nam Phu	M3	M3-15	H0000010765T00002	red
Surat Thani	Ban Na San	Nam Phu	M3	M3-16	H0000010713T00001	red
Surat Thani	Ban Na San	Nam Phu	M3	M3-17	H0000010723T00002	green
Surat Thani	Ban Na San	Nam Phu	M3	M3-18	H0000010731T00002	green
Surat Thani	Ban Na San	Nam Phu	M4	M4-13	H0000010516T00001	red
Surat Thani	Ban Na San	Nam Phu	M4	M4-14	n.d.	green
Surat Thani	Ban Na San	Nam Phu	M4	M4-15	H0000010435T00001	red
Surat Thani	Ban Na San	Nam Phu	M4	M4-16	H0000010530T00001	red
Surat Thani	Ban Na San	Nam Phu	M4	M4-17	n.d.	red
Surat Thani	Ban Na San	Nam Phu	M4	M4-18	H0000010461T00001	green
Surat Thani	Ban Na San	Nam Phu	M4	M4-19	H0000010437T00001	green
Surat Thani	Ban Na San	Nam Phu	M4	M4-20	H0000010305T00001	red
Surat Thani	Ban Na San	Nam Phu	M4	M4-21	H0000010546T00001	red
Surat Thani	Ban Na San	Nam Phu	M4	M4-22	n.d.	green
Surat Thani	Ban Na San	Nam Phu	M5	M5-18	H0000010450T00001	red
Surat Thani	Ban Na San	Nam Phu	M5	M5-19	H0000010523T00001	green
Surat Thani	Ban Na San	Nam Phu	M5	M5-20	H0000010452T00003	red
Surat Thani	Ban Na San	Nam Phu	M5	M5-21	H0000010502T00001	red
Surat Thani	Ban Na San	Nam Phu	M5	M5-22	H0000010455T00001	red
Surat Thani	Ban Na San	Nam Phu	M5	M5-23	H0000010568T00002	green
Surat Thani	Ban Na San	Nam Phu	M5	M5-24	H0000010444T00001	red
Surat Thani	Ban Na San	Nam Phu	M6	M6-22	H0000010322T00001	green
Surat Thani	Ban Na San	Nam Phu	M6	M6-23	H0000010323T00001	green
Surat Thani	Ban Na San	Nam Phu	M6	M6-24	H0000010267T00002	green

Surat Thani	Ban Na San	Nam Phu	M6	M6-25	H0000010347T00001	red

[a]The numbers (M1-M6) indicate the location of village;

[b]Sample code shows the number of samples, collected in each village.

[c]QR code is generated and established by the NCS, ONCB.

n.d.: no data.

Table S2. Information relating to kratom plant samples which were collected during October 2019.

Province	District	Subdistrict	Village	Sample code	QR code	Leaf vein color	Girth (cm)	Origin of plant	Age (years)
Surat Thani	Ban Na San	Nam Phu	M1	M1-3	H0000011525T00001	green	20	grafting	4 to 5
Surat Thani	Ban Na San	Nam Phu	M1	M1-4	H0000011596T00003	green	35	grafting	8 to 9
Surat Thani	Ban Na San	Nam Phu	M1	M1-5	H0000011527T00002	green	30	seedling	6 to 7
Surat Thani	Ban Na San	Nam Phu	M1	M1-6	H0000011590T00001	green	43.18	seedling	7
Surat Thani	Ban Na San	Nam Phu	M1	M1-7	H0000011467T00003	red	57	grafting	8 to 9
Surat Thani	Ban Na San	Nam Phu	M1	M1-8	H0000011505T00001	green	68.58	seedling	10
Surat Thani	Ban Na San	Nam Phu	M1	M1-9	H0000010547T00002	red	16	grafting	10
Surat Thani	Ban Na San	Nam Phu	M1	M1-10	H0000011592T00003	green	20	seedling	5 to 6
Surat Thani	Ban Na San	Nam Phu	M2	M2-2	H0000010623T00003	red	33	grafting	8
Surat Thani	Ban Na San	Nam Phu	M2	M2-3	H0000010449T00005	green	31.75	seedling	8
Surat Thani	Ban Na San	Nam Phu	M2	M2-4	H0000010620T00003	green	32	seedling	4
Surat Thani	Ban Na San	Nam Phu	M2	M2-5	H0000010489T00002	green	38	grafting	5
Surat Thani	Ban Na San	Nam Phu	M2	M2-6	H0000010662T00016	green	95.55	grafting	12
Surat Thani	Ban Na San	Nam Phu	M2	M2-7	H0000010656T00001	green	40	grafting	8
Surat Thani	Ban Na San	Nam Phu	M2	M2-8	H0000010659T00005	red	53	seedling	15
Surat Thani	Ban Na San	Nam Phu	M2	M2-9	H0000010610T00003	red	20	grafting	5
Surat Thani	Ban Na San	Nam Phu	M2	M2-10	H0000010613T00001	red	15	seedling	4
Surat Thani	Ban Na San	Nam Phu	M3	M3-6	H0000010708T00002	red	31	grafting	4
Surat Thani	Ban Na San	Nam Phu	M3	M3-7	H0000010767T00003	green	40	seedling	n.d.
Surat Thani	Ban Na San	Nam Phu	M3	M3-8	H0000010765T00002	red	36	seedling	4
Surat Thani	Ban Na San	Nam Phu	M3	M3-9	H0000010713T00002	red	41	cutting	4
Surat Thani	Ban Na San	Nam Phu	M3	M3-10	H0000010723T00002	red	28	seedling	3
Surat Thani	Ban Na San	Nam Phu	M3	M3-11	H0000010731T00002	green	40	seedling	3
Surat Thani	Ban Na San	Nam Phu	M3	M3-12	H0000010743T00001	green	31	seedling	3
Surat Thani	Ban Na San	Nam Phu	M3	M3-13	H0000010703T00001	green	17.78	seedling	7
Surat Thani	Ban Na San	Nam Phu	M4	M4-8	H0000010383T00001	red	28.5	seedling	12

Surat Thani	Ban Na San	Nam Phu	M4	M4-9	H0000010551T00001	green	55.83	seedling	n.d.
Surat Thani	Ban Na San	Nam Phu	M4	M4-10	H0000010516T00001	red	61	grafting	22
Surat Thani	Ban Na San	Nam Phu	M4	M4-11	H0000010481T00001	green	80	seedling	60
Surat Thani	Ban Na San	Nam Phu	M4	M4-12	H0000010530T00001	red	20.5	grafting	10
Surat Thani	Ban Na San	Nam Phu	M5	M5-11	H0000010450T00001	green	86	seedling	12
Surat Thani	Ban Na San	Nam Phu	M5	M5-12	H0000010523T00001	red	42	grafting	5
Surat Thani	Ban Na San	Nam Phu	M5	M5-13	H0000010444T00002	green	10.16	seedling	4
Surat Thani	Ban Na San	Nam Phu	M5	M5-14	H0000010452T00003	green	66	seedling	11
Surat Thani	Ban Na San	Nam Phu	M5	M5-15	H0000010502T00003	green	35.56	seedling	9
Surat Thani	Ban Na San	Nam Phu	M5	M5-16	H0000010568T00002	green	85	grafting	7
Surat Thani	Ban Na San	Nam Phu	M5	M5-17	H0000010444T00001	green	62	seedling	24
Surat Thani	Ban Na San	Nam Phu	M6	M6-14	H0000010357T00003	green	39.2	seedling	8
Surat Thani	Ban Na San	Nam Phu	M6	M6-15	H0000010324T00001	red	49.2	seedling	10
Surat Thani	Ban Na San	Nam Phu	M6	M6-16	H0000010339T00003	red	37.5	seedling	6
Surat Thani	Ban Na San	Nam Phu	M6	M6-17	H0000010241T00003	red	28.2	seedling	4
Surat Thani	Ban Na San	Nam Phu	M6	M6-18	H0000010323T00001	green	22.5	seedling	3
Surat Thani	Ban Na San	Nam Phu	M6	M6-19	H0000010267T00005	green	16	seedling	4
Surat Thani	Ban Na San	Nam Phu	M6	M6-20	H0000010347T00001	green	55.83	grafting	7
Surat Thani	Ban Na San	Nam Phu	M6	M6-21	H0000010322T00001	red	51.3	seedling	7

Table S3. Set 2 of samples, collected from the southern, northern and central regions, provided by the Narcotic Crop Survey and Monitoring Institute (NCS), the Office of Narcotic Control Board (ONCB).

Province	District	Subdistrict	Village[a]	Sample code[b]	QR code[c]	Leaf vein color
Chiangmai	Muang Chiangmai	Chang Phueak	M5	CM-PS1	H0000000000000001	green
Chiangmai	Maetaeng	San Mahaphon	M3	CM-SM1	n.d.	n.d.
Chumphon	Muang Chumphon	Bang Mak	M6	C-BM1	H0000005676T00001	green
Chumphon	Muang Chumphon	Bang Mak	M6	C-BM2	H0000004203T00001	green
Chumphon	Muang Chumphon	Bang Mak	M6	C-BM3	H0000005460T00001	green
Chumphon	Muang Chumphon	Bang Mak	M6	C-BM4	H0000005985T00001	green
Chumphon	Muang Chumphon	Bang Mak	M6	C-BM5	H0000005975T00002	red
Chumphon	Muang Chumphon	Bang Mak	M6	C-BM6	H0000005651T00003	red
Chumphon	Muang Chumphon	Bang Mak	M6	C-BM7	H0000004261T00001	green
Chumphon	Muang Chumphon	Bang Mak	M6	C-BM8	H0000005592T00001	green
Chumphon	Muang Chumphon	Bang Mak	M6	C-BM9	H0000005614T00001	red
Chumphon	Muang Chumphon	Bang Mak	M6	C-BM10	H0000005634T00001	green
Lamphun	Muang Lamphun	Pratu Pa	M5	LP-PL1	n.d.	n.d.
Lamphun	Muang Lamphun	Rimping	M2	LP-RL1	n.d.	n.d.
Lamphun	Muang Lamphun	Rimping	M4	LP-RL2	n.d.	n.d.
Nakhon Si Thammarat	Tham Phannara	Khlong Sae	M1	N-KT1	H0000006727T00001	green
Nakhon Si Thammarat	Tham Phannara	Khlong Sae	M2	N-KT2	H0000004589T00001	n.d.
Nakhon Si Thammarat	Tham Phannara	Khlong Sae	M3	N-KT3	H0000008365T00001	green
Nakhon Si Thammarat	Tham Phannara	Khlong Sae	M4	N-KT4	H0000007138T00001	green
Nakhon Si Thammarat	Tham Phannara	Khlong Sae	M5	N-KT5	H0000005629T00001	red
Nakhon Si Thammarat	Tham Phannara	Khlong Sae	M5	N-KT6	H0000004893T00001	red
Nakhon Si Thammarat	Tham Phannara	Khlong Sae	M6	N-KT7	H0000006734T00001	red
Nakhon Si Thammarat	Tham Phannara	Khlong Sae	M6	N-KT8	H0000006776T00002	red
Nakhon Si Thammarat	Tham Phannara	Khlong Sae	M7	N-KT9	H0000006260T00001	n.d.

Nakhon Si Thammarat	Tham Phannara	Khlong Sae	M8	N-KT10	H0000006080T00002	green
Nonthaburi	Sai Noi	Sai Yai	M10	NT-SS1	H0000000100T00002	green
Nonthaburi	Sai Noi	Sai Yai	M10	NT-SS2	H0000000294T00001	green
Nonthaburi	Sai Noi	Sai Yai	M10	NT-SS3	H0000000316T00004	green
Nonthaburi	Sai Noi	Sai Yai	M10	NT-SS4	H0000000335T00002	green
Nonthaburi	Sai Noi	Sai Yai	M10	NT-SS5	H0000000227T00002	green
Nonthaburi	Sai Noi	Sai Yai	M10	NT-SS6	H0000000262T00002	green
Nonthaburi	Sai Noi	Sai Yai	M10	NT-SS7	H0000000159T00003	green
Nonthaburi	Sai Noi	Sai Yai	M10	NT-SS8	H0000000132T00012	green
Nonthaburi	Sai Noi	Sai Yai	M10	NT-SS9	H0000000367T00003	green
Nonthaburi	Sai Noi	Sai Yai	M10	NT-SS10	H0000000258T00001	green
Pathum Thani	Khlong Luang	Khlong Sam	M12	PT-KK1	H0000000091T00003	green
Pathum Thani	Khlong Luang	Khlong Sam	M12	PT-KK2	H0000000121T00002	green
Pathum Thani	Khlong Luang	Khlong Sam	M12	PT-KK3	H0000000379T00001	green
Pathum Thani	Khlong Luang	Khlong Sam	M12	PT-KK4	H0000000095T00001	green
Pathum Thani	Khlong Luang	Khlong Sam	M12	PT-KK5	H0000000255T00001	green
Pathum Thani	Khlong Luang	Khlong Sam	M12	PT-KK6	H0000000127T00014	green
Pathum Thani	Khlong Luang	Khlong Sam	M12	PT-KK7	H0000000115T00003	red
Pathum Thani	Khlong Luang	Khlong Sam	M12	PT-KK8	H0000000141T00001	green
Pathum Thani	Khlong Luang	Khlong Sam	M12	PT-KK9	H0000000349T00002	green
Pathum Thani	Khlong Luang	Khlong Sam	M12	PT-KK10	H0000000208T00001	red
Phangnga	Thai Mueang	Lampi	M1	PH-LT1	H0000005305T00001	red
Phangnga	Thai Mueang	Lampi	M1	PH-LT2	H0000005466T00001	red
Phangnga	Thai Mueang	Lampi	M2	PH-LT3	H0000004491T00001	red
Phangnga	Thai Mueang	Lampi	M3	PH-LT4	H0000006444T00001	green
Phangnga	Thai Mueang	Lampi	M3	PH-LT5	H0000008303T00001	red
Phangnga	Thai Mueang	Lampi	M4	PH-LT6	H0000005569T00002	green
Phangnga	Thai Mueang	Lampi	M5	PH-LT7	H0000006799T00001	green

Phangnga	Thai Mueang	Lampi	M5	PH-LT8	H0000007326T00001	green
Phangnga	Thai Mueang	Lampi	M6	PH-LT9	H0000005838T00001	n.d.
Phangnga	Thai Mueang	Lampi	M7	PH-LT10	H0000006106T00001	green
Phatthalung	Srinakarin	Lamsin	M9	P-LS1	H0000001334T00001	green
Phatthalung	Srinakarin	Lamsin	M4	P-LS2	H0000000782T00001	red
Phatthalung	Srinakarin	Lamsin	M5	P-LS3	H0000001154T00001	green
Phatthalung	Srinakarin	Lamsin	M3	P-LS4	H0000000840T00001	green
Phatthalung	Srinakarin	Lamsin	M1	P-LS5	n.d.	red
Phatthalung	Srinakarin	Lamsin	M3	P-LS6	n.d.	green
Phatthalung	Srinakarin	Lamsin	M8	P-LS7	H0000001029T00002	green
Phatthalung	Srinakarin	Lamsin	M6	P-LS8	H0000001060T00002	green
Phatthalung	Srinakarin	Lamsin	M2	P-LS9	H0000000909T00001	green
Phatthalung	Srinakarin	Lamsin	M7	P-LS10	H0000001275T00001	green
Phetchabun	Muang Phetchabun	Wang Chompoo	M1	PB-WM1	H0000011695T00015	red
Phetchabun	Muang Phetchabun	Wang Chompoo	M2	PB-WM2	H00000117T00004	red
Phetchabun	Muang Phetchabun	Wang Chompoo	M4	PB-WM3	H0000011629T00001	green
Phetchabun	Muang Phetchabun	Wang Chompoo	M4	PB-WM4	H0000011620T00001	green
Phetchabun	Muang Phetchabun	Wang Chompoo	M5	PB-WM5	H0000011779T00001	red
Phetchabun	Muang Phetchabun	Wang Chompoo	M6	PB-WM6	H0000011815T00002	green
Phetchabun	Muang Phetchabun	Wang Chompoo	M7	PB-WM7	n.d.	n.d.
Phetchabun	Muang Phetchabun	Wang Chompoo	M7	PB-WM8	H0000011786T00001	red
Phetchabun	Muang Phetchabun	Wang Chompoo	M8	PB-WM9	n.d.	green
Phetchabun	Muang Phetchabun	Wang Chompoo	M10	PB-WM10	H0000011830T00001	green
Phetchabun	Muang Phetchabun	Wang Chompoo	M11	PB-WM11	H0000011657T00001	green
Phetchabun	Muang Phetchabun	Wang Chompoo	M12	PB-WM12	H0000011723T00001	red
Phetchabun	Muang Phetchabun	Wang Chompoo	M12	PB-WM13	H0000011725T00001	red
Phetchabun	Muang Phetchabun	Wang Chompoo	M13	PB-WM14	n.d.	green
Phetchabun	Muang Phetchabun	Wang Chompoo	M14	PB-WM15	H0000011725T00001	green

Phetchabun	Muang Phetchabun	Wang Chompoo	M14	PB-WM16	n.d.	green
Prachuap Khiri Khan	Bang Saphan Noi	Chang Raek	M3	PJ-CB1	H0000011074T00001	n.d.
Prachuap Khiri Khan	Bang Saphan Noi	Chang Raek	M6	PJ-CB2	H0000001133T00001	n.d.
Prachuap Khiri Khan	Bang Saphan Noi	Chang Raek	M8	PJ-CB3	H0000010941T00001	n.d.
Prachuap Khiri Khan	Bang Saphan Noi	Chang Raek	M2	PJ-CB4	H0000011212T00002	n.d.
Prachuap Khiri Khan	Bang Saphan Noi	Chang Raek	M7	PJ-CB5	H0000011036T00001	n.d.
Prachuap Khiri Khan	Bang Saphan Noi	Chang Raek	M8	PJ-CB6	H0000011379T00002	n.d.
Prachuap Khiri Khan	Bang Saphan Noi	Chang Raek	M1	PJ-CB7	H0000010918T00001	n.d.
Prachuap Khiri Khan	Bang Saphan Noi	Chang Raek	M5	PJ-CB8	H0000011231T00002	n.d.
Prachuap Khiri Khan	Bang Saphan Noi	Chang Raek	M7	PJ-CB9	H0000010978T00002	n.d.
Prachuap Khiri Khan	Bang Saphan Noi	Chang Raek	M4	PJ-CB10	H0000011106T00001	n.d.
Ranong	Laun	Laun Nuea	M1	R-LL1	H0000001374T00003	green
Ranong	Laun	Laun Nuea	M1	R-LL2	H0000001381T00002	green
Ranong	Laun	Laun Nuea	M2	R-LL3	H0000004067T00004	red
Ranong	Laun	Laun Nuea	M2	R-LL4	H0000004063T00001	red
Ranong	Laun	Laun Nuea	M3	R-LL5	H0000004028T00002	red
Ranong	Laun	Laun Nuea	M3	R-LL6	H0000006529T00003	red
Ranong	Laun	Laun Nuea	M4	R-LL7	H0000004233T00002	red
Ranong	Laun	Laun Nuea	M4	R-LL8	H0000003523T00001	green
Ranong	Laun	Laun Nuea	M5	R-LL9	H0000001436T00001	red
Ranong	Laun	Laun Nuea	M5	R-LL10	H0000001400T00001	red
Ranong	Laun	Laun Nuea	M4	R-LL11	H0000003526T00001	green
Surat Thani	Ban Na San	Khlong Prap	M1	S-KPN1	H0000006172T00001	red
Surat Thani	Ban Na San	Khlong Prap	M1	S-KPN2	H0000006254T00001	green
Surat Thani	Ban Na San	Khlong Prap	M2	S-KPN3	H0000001837T00001	red
Surat Thani	Ban Na San	Khlong Prap	M2	S-KPN4	H0000001757T00001	red
Surat Thani	Ban Na San	Khlong Prap	M3	S-KPN5	H0000002018T00001	green
Surat Thani	Ban Na San	Khlong Prap	M3	S-KPN6	H0000002024T00001	green

Surat Thani	Ban Na San	Khlong Prap	M4	S-KPN7	H0000001802T00001	red
Surat Thani	Ban Na San	Khlong Prap	M4	S-KPN8	H0000001816T00001	red
Surat Thani	Ban Na San	Khlong Prap	M5	S-KPN9	H0000006514T00002	red
Surat Thani	Ban Na San	Khlong Prap	M5	S-KPN10	H0000008464T00001	red
Surat Thani	Ban Na San	Khuan Si	M1	S-KSN1	H0000002999T00003	green
Surat Thani	Ban Na San	Khuan Si	M1	S-KSN2	H0000003026T00001	green
Surat Thani	Ban Na San	Khuan Si	M2	S-KSN3	H0000001895T00001	green
Surat Thani	Ban Na San	Khuan Si	M3	S-KSN4	H0000002550T00002	red
Surat Thani	Ban Na San	Khuan Si	M4	S-KSN5	H0000002111T00001	green
Surat Thani	Ban Na San	Khuan Si	M5	S-KSN6	H0000002377T00001	green
Surat Thani	Ban Na San	Khuan Si	M6	S-KSN7	H0000001756T00002	green
Surat Thani	Ban Na San	Khuan Si	M7	S-KSN8	H0000001687T00001	green
Surat Thani	Ban Na San	Khuan Si	M7	S-KSN9	H0000001616T00001	red
Surat Thani	Ban Na San	Khuan Si	M8	S-KSN10	H0000008449T00001	green
Surat Thani	Ban Na San	Kuan Suban	M1	S-KBN1	H0000005799T00003	n.d.
Surat Thani	Ban Na San	Kuan Suban	M2	S-KBN2	H0000008444T00001	n.d.
Surat Thani	Ban Na San	Kuan Suban	M2	S-KBN3	H0000008666T00002	n.d.
Surat Thani	Ban Na San	Kuan Suban	M3	S-KBN4	H0000006842T00001	n.d.
Surat Thani	Ban Na San	Kuan Suban	M3	S-KBN5	H0000009602T00001	n.d.
Surat Thani	Ban Na San	Kuan Suban	M4	S-KBN6	H0000010190T00001	n.d.
Surat Thani	Ban Na San	Kuan Suban	M4	S-KBN7	H0000008875T00001	n.d.
Surat Thani	Ban Na San	Kuan Suban	M5	S-KBN8	H0000005028T00001	n.d.
Surat Thani	Ban Na San	Kuan Suban	M6	S-KBN9	H0000003865T00001	n.d.
Surat Thani	Ban Na San	Kuan Suban	M7	S-KBN10	H0000008826T00001	n.d.
Surat Thani	Ban Na San	Lamphun	M1	S-LN1	H0000003744T00002	green
Surat Thani	Ban Na San	Lamphun	M1	S-LN2	H0000003713T00001	green
Surat Thani	Ban Na San	Lamphun	M2	S-LN3	H0000006690T00002	n.d.
Surat Thani	Ban Na San	Lamphun	M3	S-LN4	H0000004817T00002	green

Surat Thani	Ban Na San	Lamphun	M3	S-LN5	H0000004857T00002	green
Surat Thani	Ban Na San	Lamphun	M4	S-LN6	H0000005025T00001	n.d.
Surat Thani	Ban Na San	Lamphun	M5	S-LN7	H0000003317T00001	green
Surat Thani	Ban Na San	Lamphun	M6	S-LN8	H0000003418T00001	red
Surat Thani	Ban Na San	Lamphun	M7	S-LN9	H0000009966T00002	n.d.
Surat Thani	Ban Na San	Lamphun	M7	S-LN10	H0000002774T00003	n.d.
Surat Thani	Ban Na San	Nam Phu	M1	S-NN1	H0000011503T00004	green
Surat Thani	Ban Na San	Nam Phu	M1	S-NN2	H0000011531T00001	green
Surat Thani	Ban Na San	Nam Phu	M2	S-NN3	H0000010561T00001	green
Surat Thani	Ban Na San	Nam Phu	M2	S-NN4	H0000010446T00003	green
Surat Thani	Ban Na San	Nam Phu	M3	S-NN5	H0000010766T00001	green
Surat Thani	Ban Na San	Nam Phu	M4	S-NN6	H0000010458T00001	green
Surat Thani	Ban Na San	Nam Phu	M4	S-NN7	H0000010546T00002	green
Surat Thani	Ban Na San	Nam Phu	M5	S-NN8	H0000010479T00003	red
Surat Thani	Ban Na San	Nam Phu	M6	S-NN9	H0000010221T00001	green
Surat Thani	Ban Na San	Nam Phu	M6	S-NN10	H0000010269T00002	green
Surat Thani	Ban Na San	Nasan municipality		S-MN1	H0000009892T00001	n.d.
Surat Thani	Ban Na San	Nasan municipality		S-MN2	H0000009283T00001	green
Surat Thani	Ban Na San	Nasan municipality		S-MN3	H0000005826T00003	green
Surat Thani	Ban Na San	Nasan municipality		S-MN4	H0000007449T00001	red
Surat Thani	Ban Na San	Nasan municipality		S-MN5	H0000009522T00002	red
Surat Thani	Ban Na San	Nasan municipality		S-MN6	H0000008774T00001	red
Surat Thani	Ban Na San	Nasan municipality		S-MN7	H0000008984T00001	red

Surat Thani	Ban Na San	Nasan municipality		S-MN8	H0000008622T00001	green
Surat Thani	Ban Na San	Nasan municipality		S-MN9	H0000007801T00001	green
Surat Thani	Ban Na San	Nasan municipality		S-MN10	H0000009530T00001	green
Surat Thani	Ban Na San	Permphun Sub	M1	S-PPN1	H0000003248T00001	n.d.
Surat Thani	Ban Na San	Permphun Sub	M1	S-PPN2	H0000009803T00001	n.d.
Surat Thani	Ban Na San	Permphun Sub	M2	S-PPN3	H0000008093T00001	n.d.
Surat Thani	Ban Na San	Permphun Sub	M3	S-PPN4	H0000004990T00003	n.d.
Surat Thani	Ban Na San	Permphun Sub	M3	S-PPN5	H0000004941T00003	n.d.
Surat Thani	Ban Na San	Permphun Sub	M4	S-PPN6	H0000008160T00002	n.d.
Surat Thani	Ban Na San	Permphun Sub	M4	S-PPN7	H0000008169T00001	n.d.
Surat Thani	Ban Na San	Permphun Sub	M5	S-PPN8	H0000003380T00001	n.d.
Surat Thani	Ban Na San	Permphun Sub	M6	S-PPN9	H0000006428T00001	n.d.
Surat Thani	Ban Na San	Permphun Sub	M6	S-PPN10	H0000009101T00002	n.d.
Surat Thani	Ban Na San	Phru Phi	M1	S-PN1	H0000002917T00004	green
Surat Thani	Ban Na San	Phru Phi	M2	S-PN2	H0000003134T00001	green
Surat Thani	Ban Na San	Phru Phi	M3	S-PN3	H0000003200T00002	green
Surat Thani	Ban Na San	Phru Phi	M3	S-PN4	H0000003192T00002	green
Surat Thani	Ban Na San	Phru Phi	M4	S-PN5	H0000003049T00001	red
Surat Thani	Ban Na San	Phru Phi	M5	S-PN6	H0000002297T00001	red
Surat Thani	Ban Na San	Phru Phi	M5	S-PN7	H0000001393T00001	red
Surat Thani	Ban Na San	Phru Phi	M6	S-PN8	H0000002825T00001	red
Surat Thani	Ban Na San	Phru Phi	M7	S-PN9	H0000003506T00001	red
Surat Thani	Ban Na San	Phru Phi	M7	S-PN10	H0000009631T00001	green
Surat Thani	Ban Na San	Tha Chi	M1	S-TCN1	H0000002566T00002	red
Surat Thani	Ban Na San	Tha Chi	M1	S-TCN2	H0000002465T00002	red
Surat Thani	Ban Na San	Tha Chi	M2	S-TCN3	H0000003477T00001	red

Surat Thani	Ban Na San	Tha Chi	M2	S-TCN4	H0000002148T00001	red
Surat Thani	Ban Na San	Tha Chi	M3	S-TCN5	H0000002516T00001	green
Surat Thani	Ban Na San	Tha Chi	M3	S-TCN6	H0000002826T00003	green
Surat Thani	Ban Na San	Tha Chi	M4	S-TCN7	H0000004007T00002	green
Surat Thani	Ban Na San	Tha Chi	M4	S-TCN8	H0000003734T00002	green
Surat Thani	Ban Na San	Tha Chi	M5	S-TCN9	H0000002638T00003	red
Surat Thani	Ban Na San	Tha Chi	M6	S-TCN10	H0000003647T00001	red
Surat Thani	Ban Na San	Thung Tao	M1	S-TTN1	H0000007661T00002	red
Surat Thani	Ban Na San	Thung Tao	M1	S-TTN2	H0000006964T00001	red
Surat Thani	Ban Na San	Thung Tao	M2	S-TTN3	H0000004870T00001	green
Surat Thani	Ban Na San	Thung Tao	M2	S-TTN4	H0000005172T00002	green
Surat Thani	Ban Na San	Thung Tao	M3	S-TTN5	H0000005733T00003	green
Surat Thani	Ban Na San	Thung Tao	M3	S-TTN6	H0000005701T00002	green
Surat Thani	Ban Na San	Thung Tao	M4	S-TTN7	H0000003949T00002	green
Surat Thani	Ban Na San	Thung Tao	M4	S-TTN8	H0000003540T00002	green
Surat Thani	Ban Na San	Thung Tao	M5	S-TTN9	H0000006889T00001	green
Surat Thani	Ban Na San	Thung Tao	M5	S-TTN10	H0000003742T00002	green
Surat Thani	Ban Na San	Thung Tao Mai	M1	S-TMN1	H0000003940T00001	red
Surat Thani	Ban Na San	Thung Tao Mai	M2	S-TMN2	H0000007422T00001	red
Surat Thani	Ban Na San	Thung Tao Mai	M3	S-TMN3	H0000005395T00001	red
Surat Thani	Ban Na San	Thung Tao Mai	M3	S-TMN4	H0000004570T00001	green
Surat Thani	Ban Na San	Thung Tao Mai	M4	S-TMN5	H0000003759T00002	green
Surat Thani	Ban Na San	Thung Tao Mai	M4	S-TMN6	H0000009638T00001	green
Surat Thani	Ban Na San	Thung Tao Mai	M5	S-TMN7	H0000007289T00001	n.d.
Surat Thani	Ban Na San	Thung Tao Mai	M6	S-TMN8	H0000003881T00001	red
Surat Thani	Ban Na San	Thung Tao Mai	M7	S-TMN9	H0000007040T00001	green
Surat Thani	Ban Na San	Thung Tao Mai	M8	S-TMN10	H0000006626T00001	green
Surat Thani	Ban Na San	Thung Tao Mai	M3	S-TMN11	H0000004620T00001	green

Trang	Huai yot	Nai Tao	M3	T-NH1	H0000000582T00002	green
Trang	Huai yot	Nai Tao	M2	T-NH2	H0000000488T00002	green
Trang	Huai yot	Nai Tao	M3	T-NH3	H0000000112T00001	green
Trang	Huai yot	Nai Tao	M1	T-NH4	H0000000461T00003	green
Trang	Huai yot	Nai Tao	M2	T-NH5	H0000000514T00001	red
Trang	Huai yot	Nai Tao	M1	T-NH6	H0000000477T00001	green
Trang	Huai yot	Nai Tao	M3	T-NH7	H0000001112T00002	green
Trang	Huai yot	Nai Tao	M1	T-NH8	H0000000397T00001	green
Trang	Huai yot	Nai Tao	M4	T-NH9	H0000000672T00001	red
Trang	Huai yot	Nai Tao	M4	T-NH10	H0000000714T00003	green

[a]The numbers indicate the location of village;

[b]Sample code shows the number of samples, collected in each village.

[c]QR code is generated and established by the NCS, ONCB.

n.d.: no data.
